# Molecular Diversity of Anesthetic Actions Is Evident in Electroencephalogram Effects in Humans and Animals

**DOI:** 10.3390/ijms22020495

**Published:** 2021-01-06

**Authors:** Sarah Eagleman, M. Bruce MacIver

**Affiliations:** Department of Anesthesiology, Perioperative and Pain Medicine, School of Medicine, Stanford University, Stanford, CA 94304, USA; saraheagleman@stanford.edu

**Keywords:** anesthetic depth, electroencephalogram, complexity, consciousness, nonlinear dynamics

## Abstract

Anesthetic agents cause unique electroencephalogram (EEG) activity resulting from actions on their diverse molecular targets. Typically to produce balanced anesthesia in the clinical setting, several anesthetic and adjuvant agents are combined. This creates challenges for the clinical use of intraoperative EEG monitoring, because computational approaches are mostly limited to spectral analyses and different agents and combinations produce different EEG responses. Thus, testing of many combinations of agents is needed to generate accurate, protocol independent analyses. Additionally, most studies to develop new computational approaches take place in young, healthy adults and electrophysiological responses to anesthetics vary widely at the extremes of age, due to physiological brain differences. Below, we discuss the challenges associated with EEG biomarker identification for anesthetic depth based on the diversity of molecular targets. We suggest that by focusing on the generalized effects of anesthetic agents on network activity, we can create paths for improved universal analyses.

## 1. The Broad Molecular Targets of Anesthetics

Anesthetic agents target and disrupt a variety of molecular receptors [1,2]. The diversity of these molecular actions has inhibited the identification of accurate, universal electrophysiological biomarkers to better monitor patients (Table 1). The large-scale, system-level disruption in brain activity is underpinned by diverse perturbations of various sleep-promoting and arousal networks in the brain. Here, we summarize the clinical challenges that impede electrophysiological biomarker discovery including anesthetics that target unique molecular cascades, multimodal general anesthesia [3], and age-dependent brain changes. We also suggest a unique opportunity to explore “final common pathway” electrophysiological biomarkers by deepening our understanding of how agents with diverse targets may converge on similar endpoints (e.g., disrupting large-scale brain activity). We review our previously published research [4,5,6,7], which highlights monitoring challenges and proposes new directions for anesthetic depth electroencephalogram (EEG) measures. Building computational tools that capture the broad network level disruptions that occur with all anesthetic agents, regardless of their molecular targets, will lead to EEG analytical tools being more predictive of anesthetic depth.

## 2. The Challenge of Finding Common Electrophysiology Biomarkers

**Figure 1:**
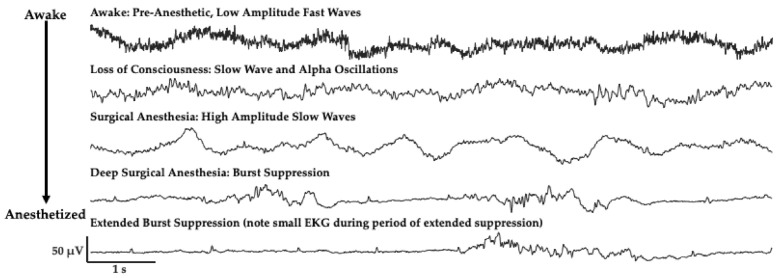
Patient electroencephalogram (EEG) signals change with increasing anesthetic depth when administered propofol, traces from frontal electrode site (F7) from a patient anesthetized with propofol. High frequency, low amplitude activity slows to include predominantly alpha and slow oscillations with anesthetic administration. Activity slows more to include a slow-wave dominant pattern, much like during natural sleep. At profound levels of unconsciousness (bottom two traces) a burst suppression pattern emerges, and suppressed periods extend as patients reach more profound levels of unconsciousness. Note the electrical activity from heart beats (electrocardiogram, EKG) which can be seen in the prolonged suppressed period.

Electroencephalogram (EEG) activity is measured using noninvasive, scalp electrodes. A single electrode contact represents a summation of tens of millions of neurons integrated over an area of 10 cm^2^ [17,18,19]. Research as early as the 1970s has noted the diversity of anesthetic agent influence on EEG signals in humans [20], and several articles have summarized and elaborated on these changes [8]. The most commonly used anesthetic agents, propofol and the halogenated ethers (e.g., sevoflurane, isoflurane), promote sleep-like electrophysiological patterns as anesthetic depth increases (Figure 1); intravenous agents primarily increase GABAergic inhibition, while volatile agents mainly depress glutamate-mediated excitation throughout the brain to produce a depression of signaling [1,21]. In addition to the loss of higher frequency activity and increase in lower frequency activity, there is also an alpha frequency (8–12 Hz) that appears in the EEG signal in unconscious states [8,22,23] (Figure 1). The appearance of a dominant alpha-rhythm is suggested to arise from disruption of thalamo-cortical communication [3,23,24]. Unlike sleep, the administration of higher doses of these anesthetic agents can drive patients to a more profound level of unconsciousness characterized by long periods of cortical suppression with punctate bursting activity (Figure 1), known as burst suppression. This suppression period extends as the level of anesthetic depth increases (Figure 1).

Given the pronounced changes in spectral activity following loss of response (LOR) using propofol and the halogenated ethers, traditional spectral analyses show a strong relationship with—and have therefore been extensively used to quantify—anesthetic depth [8,22,23].

**Figure 2:**
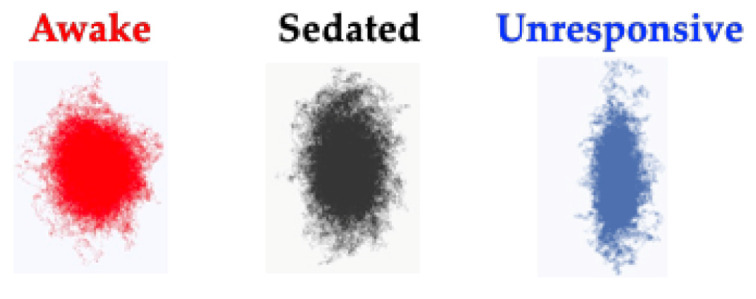
EEG attractors from rodent prefrontal cortex, showing how the geometrical shape changes with anesthesia exposure. Three-dimensional, time-delayed embeddings (attractors) change shape as rodents are exposed to deepening levels of anesthesia. Attractors flatten from inflated spheres during awake states (red), to flatter ellipsoids as anesthetic depth increases during sedation (black) and flatten even further during unresponsive (blue) states. Adapted with permission from Reference [4].

As signals slow and the brain oscillations take on more redundant and repetitive patterns, an additional way to quantify changes in the EEG signal is by calculating its complexity. A common method to calculate complexity comes from a nonlinear dynamic technique to extract dynamical attractors from the EEG signals. Here, a single EEG channel can be used to generate an attractor. An attractor is created by plotting EEG signal amplitudes at different time delays, each on a different axis. Interestingly, when this is done with electrophysiological signals from different anesthetic depths, shape changes occur [25,26]. Specifically, three dimensional attractors derived from electrophysiological signals change from inflated spheres during awake states, to flattened ellipsoids as individuals are sedated, and flatten even more during periods of unresponsiveness. Figure 2 shows these changes from extracellular local field potentials from rodent prefrontal cortex during awake (red), sedated (black), and unresponsive (blue) states. Unresponsiveness was assessed using the loss of righting reflex, a surrogate measure for loss of responsiveness in rodents [27].

**Figure 3:**
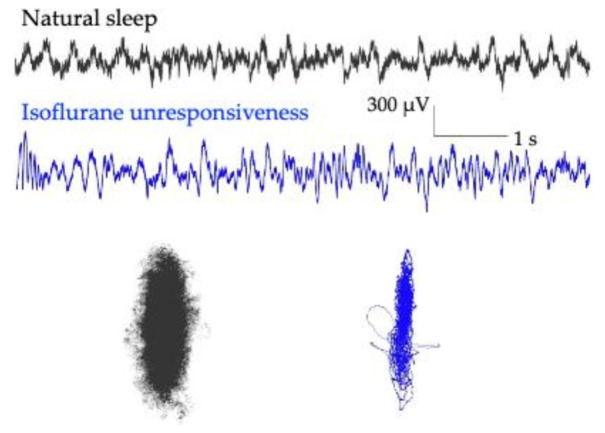
Rodent prefrontal local field potential activity and three-dimensional attractors during natural sleep and anesthesia. Local field potentials show less high frequency activity and more pronounced delta activity during anesthesia with isoflurane (blue) compared to natural sleep (black). Additionally, attractors generated from this activity show more pronounced flattening during isoflurane unresponsiveness (blue) compared to natural sleep (black), in the same animal. Adapted here with permission from Reference [4].

Sleep within the brain involves the orchestration of several sleep-promoting and arousal systems with diverse molecular targets underpinning these networks [28]. Sleep causes widespread changes in brain activity, and most species cycle through several stages of sleep. Human sleep can be subdivided into non rapid eye movement (NREM) sleep, which includes stages 1 and 2 (light sleep), and stages 3 and 4 (deep sleep or slow-wave sleep), and rapid eye movement (REM, dream) sleep. Stages 3 and 4 are the deepest level of sleep, where high amplitude delta activity predominates (Figure 3). GABAergic agents such as propofol and the halogenated ethers (e.g., isoflurane and sevoflurane) also produce a delta dominated anesthetic state (Figure 3). When local field potential signals from rodent prefrontal cortex are expressed as 3D attractors (shown in 2D Figure 3), flattening and a more ellipsoidal shape is observed when the animal is asleep; however, more dramatic shape change is observed following loss of the righting reflex with isoflurane anesthesia administration (Figure 3).

**Figure 4:**
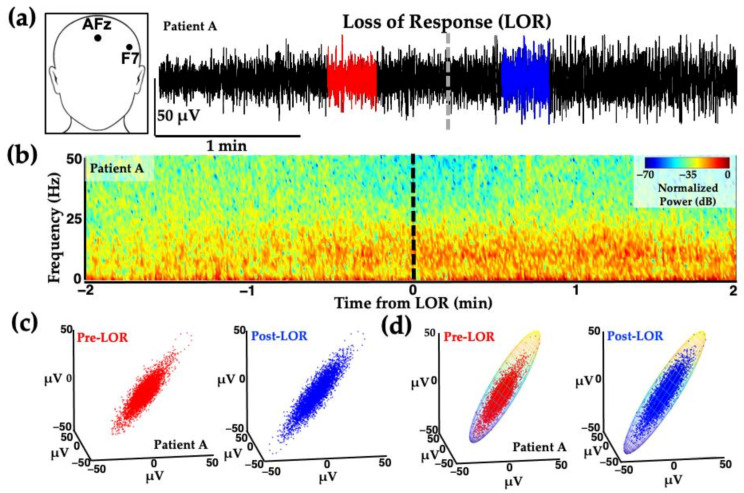
Example of frontal activity from patient EEGs before and after loss of response with propofol. (**a**) EEG activity from electrode location F7 (inset) before (red) and after (blue) loss of response (LOR, indicated with dashed line) in patient anesthetized with a slow infusion of propofol. (**b**) Normalized spectrogram of EEG activity starting from 2 min before to 2 min following LOR. (**c**) Attractors from EEG activity from patients before (red) to after (blue) LOR. Following LOR, a geometric shape change occurs where attractors become more ellipsoidal. (**d**) Same attractors from (**c**), fitted with ellipsoid solid of revolution for subsequent analysis. Reproduced with permission from Reference [7].

Similar attractor shape changes occur in human patients exposed to propofol for clinical procedures (Figure 4). EEG data shown in Figure 4 was collected from patients receiving slow infusions; the before and after clips used for analysis immediately surrounding loss of responsiveness (LOR) are not as dramatic as the effects seen with bolus infusions.

**Figure 5:**
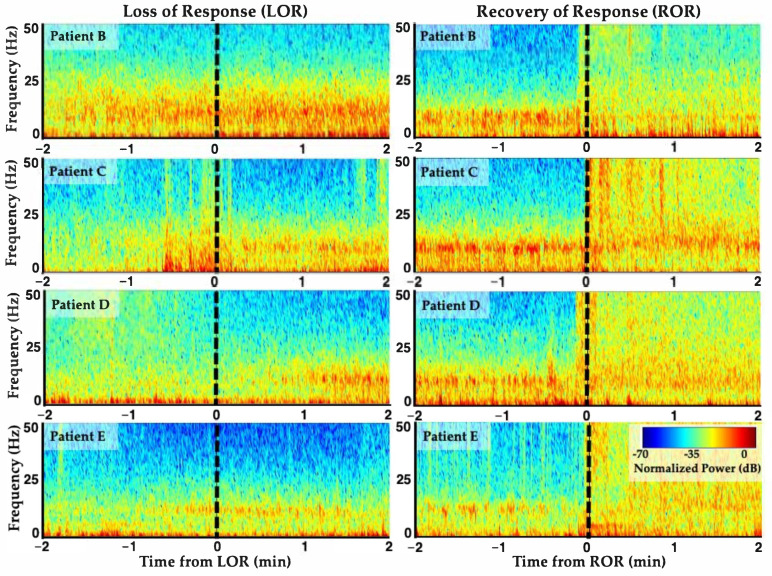
Individual variability in EEG response dynamics to propofol anesthesia. Spectrograms from four patients’ frontal EEGs surrounding loss of response (LOR) and recovery of response (ROR) with propofol anesthesia. LOR and ROR timepoints indicated with dashed lines. Decreases in high frequency activity, increases in low frequency and alpha band activity occur with LOR, and vice versa for ROR. However, individual EEG response dynamics around these time points differ between individuals. Reproduced with permission from Reference [7].

On average, when GABAergic agents are administered, there is a loss in high frequency activity, increase in low frequency activity, and higher alpha-band activity following LOR (Figure 5, left). The opposite trend occurs upon recovery of response (ROR) (Figure 5, right). These results are aligned with previous reports of propofol loss and recovery dynamics from frontal lobe sites [8,23]. Note that individual patients can have varying electrophysiological response dynamics around LOR and ROR given their individual dynamic responses to the anesthetic agents (Figure 5). Differences in response dynamics may occur because of the molecular binding dynamics of diverse agents or between individuals themselves [29,30].

**Figure 6:**
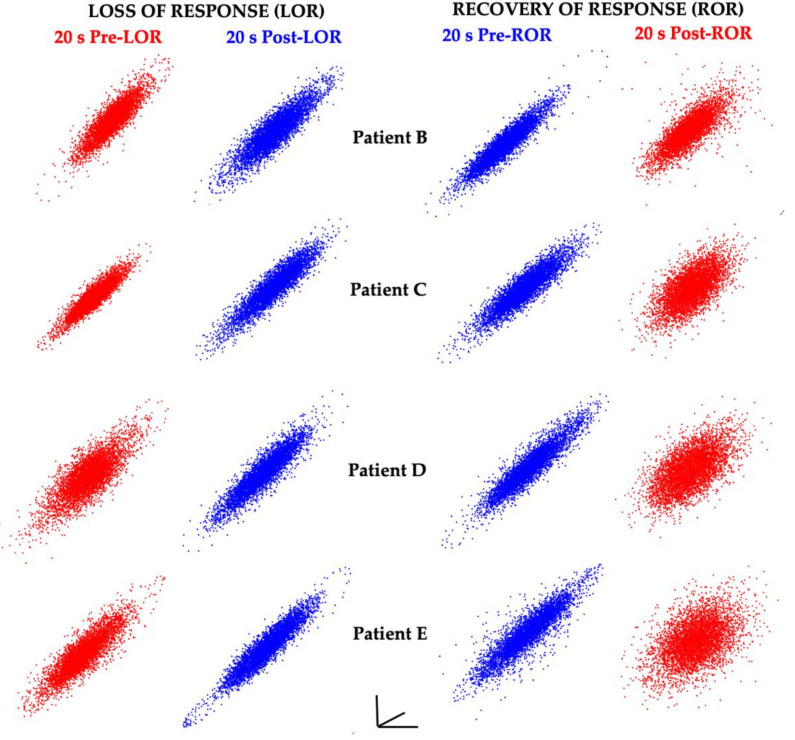
EEG attractors consistently demonstrate shape changes with changes in responsiveness. Attractors from 20 s EEG clips from four patients (the same patients as shown in Figure 5) before and after LOR and ROR when anesthetized with propofol. Awake, responsive states are shown in red, and anesthetized, unresponsive states are shown in blue. Attractors from awake, responsive states are more spherical; whereas attractors from anesthetized, unresponsive states are more ellipsoidal. Attractors are auto-scaled to illustrate the shape changes that occur with LOR and ROR. The axes are shown to demonstrate that these are 2D projections of 3D attractors. Reproduced with permission from Reference [7].

When the same time-delayed embedding procedure was applied to 20s EEG clips before and after LOR and before and after ROR, consistent shape changes were observed (Figure 6).

**Figure 7:**
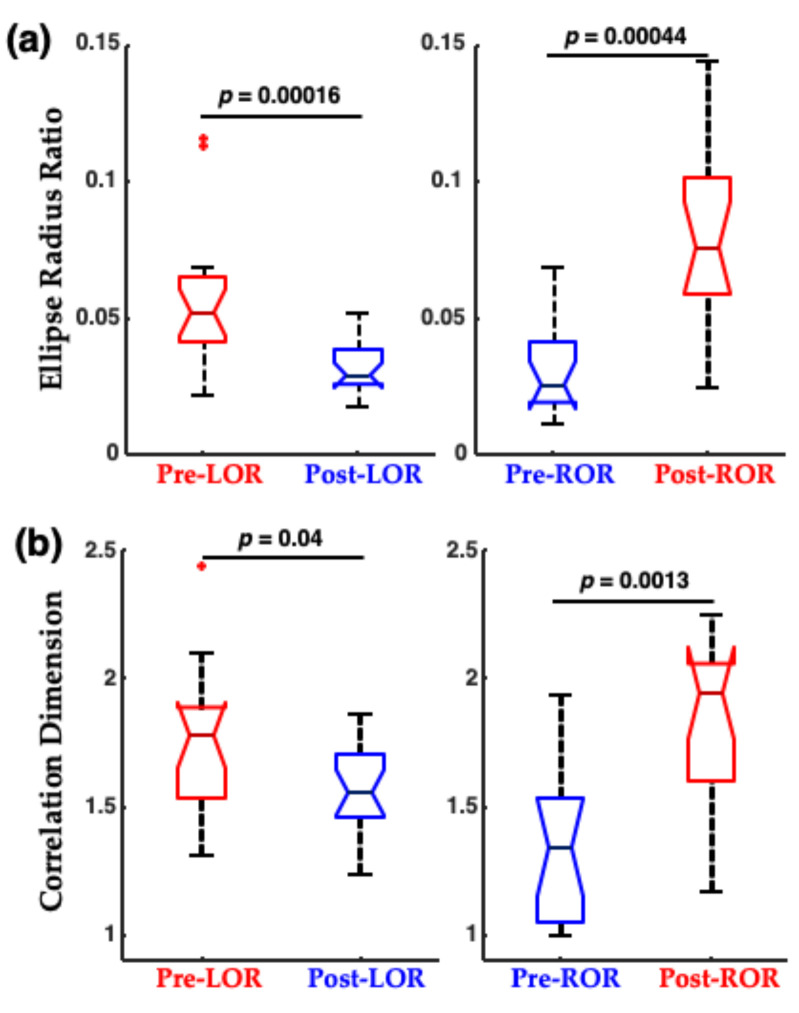
Attractor measures before and after LOR and ROR in patients exposed to propofol anesthesia. (**a**) Geometric phase–space analysis (ellipse radius ratio) significantly differs from before to after LOR, and before to after ROR. (**b**) Correlation dimension significantly differs from before to after ROR, and decreases (although not significantly in our sample) from before to after LOR. The *p*-values shown here are uncorrected. Adapted with permission from Reference [7].

When this shape change was quantified using a traditional nonlinear dynamic measure, correlation dimension (CD), and using a geometric phase–space analysis, termed the ellipse radius ratio (ERR), significant differences were observed between before and after the LOR and ROR states (Figure 7). Correlation dimension is a nonlinear dynamic technique to compute the non-integer (fractal) dimensionality of irregular objects [31,32]. For the geometric phase–space analysis, the 3D attractor was fitted with an ellipsoid solid of revolution [33], and then the ratio of the minor and major axes were computed [5,6,7]. Ratios closer to 1 occurred with more spheroidal attractors, and smaller fractions occurred with more ellipsoidal attractors. We have demonstrated that this geometric phase–space analysis is correlated with other complexity measures [6,7].

**Figure 8:**
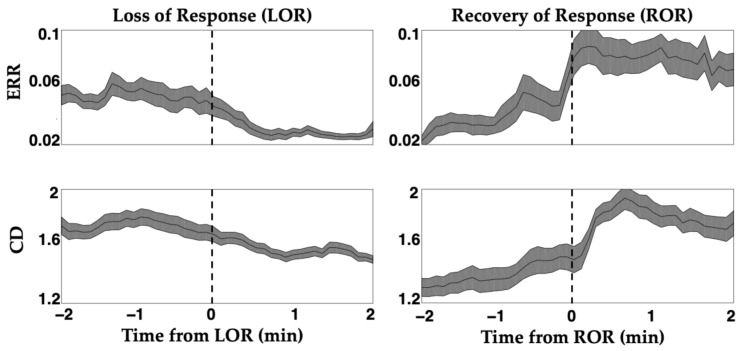
Attractor measures exhibit differing dynamics around loss and recovery of response. Induction of anesthesia and emergence from anesthesia produces an asymmetrical dynamic, known as hysteresis. Complexity measures, both the ellipse radius ratio (ERR) and the correlation dimension (CD), track these different dynamics at loss of response (LOR) and recovery of response (ROR). LOR and ROR timepoint areas are indicated with dashed lines. Dynamics are more gradual during LOR and more abrupt during ROR. Differences in dynamics observed here support existing paradigms that these brain state transitions are unique. Solid black lines represent the mean complexity values at each time point for all patients, and the shaded area is the standard error of the mean. Adapted with permission from Reference [7].

Differences in spectral content (Figure 5) and complexity measures (Figure 7) are observed between immediately after LOR and immediately before ROR. This is anticipated because the brain’s response to anesthetic agents is asymmetrical across induction and emergence. That is, the concentration of anesthetic agents is not the same when patients lose responsiveness and regain it, and their brain electrophysiological response is not the same at these two time points [34,35,36]. Interestingly, we observed similar differences in dynamics in patients exposed to propofol (Figure 8). Specifically, complexity measures exhibited more gradual changes around LOR and more abrupt changes with ROR (Figure 8).

For anesthetic agents that predominately influence GABAergic networks, we have demonstrated that complexity measures readily distinguish between the electrophysiological changes that occur before and after loss and recovery of response, in rodents [4] and humans [7]. Complexity measures capture how much the signal varies through time. Similar measures of complexity and information carried by the signal have demonstrated high utility, distinguishing between responsive and unresponsive states when using similar agents in rodents [37] and humans [38,39,40,41]. These measures capitalize on the widespread disruptions of network activity that occur with anesthesia as functional connectivity changes and brain activity becomes more synchronous and periodic [42,43,44,45,46]. These measures also capture the asymmetrical brain response to anesthetic transitions, such as that which occurs with hysteresis for GABAergic agents. However, the network modulation we have discussed only occurs in a few select agents and these agents are not usually administered alone.

## 3. Unique Anesthetic Combinations Influence Diverse Molecular Targets

Not all anesthetic agents promote sleep-like activity when administered, and often several anesthetic agents are administered in parallel to achieve balanced anesthesia. A multimodal general anesthesia approach [3] utilizes multiple agents with complementary effects to reduce the total anesthetic administered from each anesthetic class and reduce the side effects from individual agents. Monitoring multimodal general anesthesia produces further challenges, because different classes of agents have distinct molecular targets and generate unique downstream effects on the resulting EEG activity [1,2,3,8,28]. For instance, some adjuvant agents such as nitrous oxide (an NMDA antagonist), ketamine (which has many molecular targets, including NMDA receptors [47,48]), and dexmedetomidine (an alpha-2 adrenoceptor agonist) have paradoxical influences on brain activity; high frequency activity in the brain is maintained when these agents are administered, even as patients become less responsive, even when combined with other agents [8,9,49,50,51,52,53,54]. However, the complex EEG activity that is produced provides the opportunity to test computational tools that may capture network disruption generally. We tested whether complexity measures could discriminate before and after LOR and ROR when patients were administered a combination of remifentanil and nitrous oxide (N_2_O) [5].

Remifentanil is a mu-type-opioid receptor agonist and is often used as an adjuvant anesthetic agent to provide analgesia and overall decrease the administered anesthetic dosages [14]. The effects of remifentanil are potent and fast-acting, and a similar slowing of brain activity, similar to that caused by GABAergic agents, occurs with remifentanil administration [15]. Nitrous oxide (N_2_O) is an NMDA antagonist and is often used to supplement other general anesthetic agents. When co-administered with other agents, such as sevoflurane, N_2_O administration has been shown to increase slow oscillations [55]. However, N_2_O administration alone maintains [9,49,50]—and at times even enhances [10,56]—high frequency EEG activity. N_2_O addition to propofol anesthesia can increase bispectral (BIS) indices, incorrectly indicating wakefulness when patients are even more unresponsive [57].

**Figure 9:**
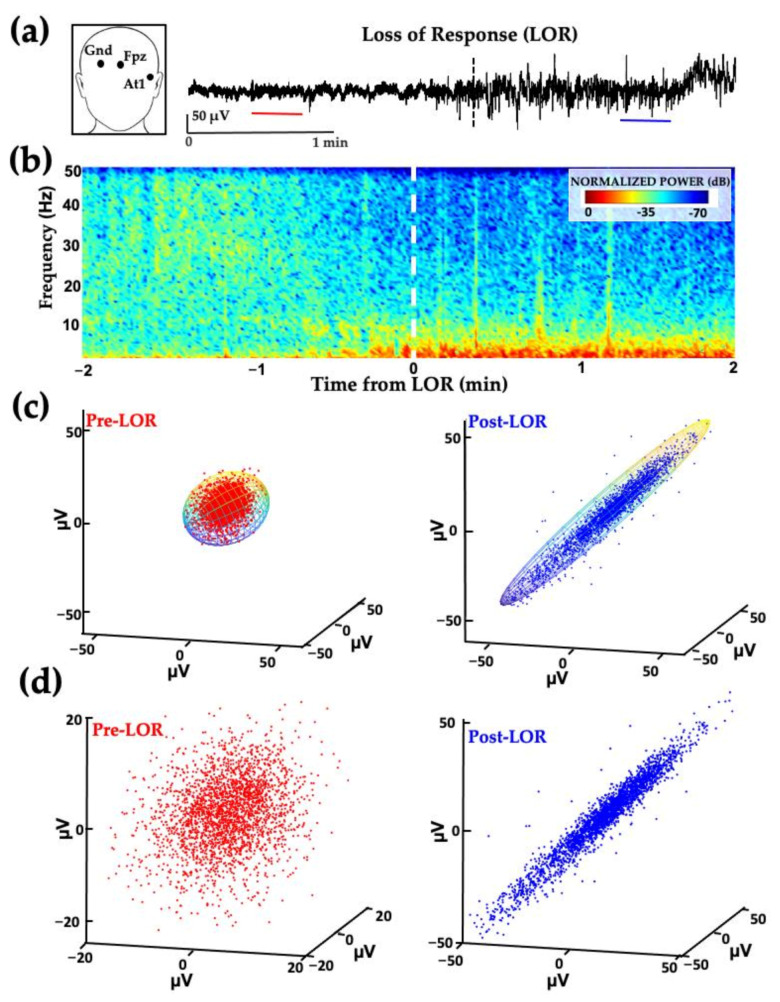
Example of patient EEG frontotemporal activity from before and after loss of response from remifentanil and nitrous oxide anesthesia. (**a**) EEG activity from electrode location At1 (inset) before and after loss of response (LOR, indicated with the dashed line) in patient anesthetized with a combination of remifentanil and 66% nitrous oxide (N_2_O). (**b**) Normalized spectrogram of EEG activity starting from 2 min before LOR to 2 min following LOR. (**c**) Attractors from 20 s EEG activity from patients before LOR (red, from red line shown in (**a**)) to after LOR (blue, from blue line shown in (**b**)) fitted with ellipsoid solid of revolution. Following LOR, a geometric shape change occurs where attractors become more ellipsoidal and flatten. (**d**) Same attractors from (**c**), auto-scaled to show geometric shape changes. Reproduced here with permission from Reference [5].

We calculated spectral and complexity measures of EEG signals from At1 (a location between the temporal and frontal lobes) in which the remifentanil blood concentrations for anesthesia were determined when used in combination with a steady background of 66% N_2_O [58]. Interestingly, slowing of the EEG activity similar to that observed by administering remifentanil alone [15] predominated, following co-administration of these agents (Figure 9). The attractor shapes also flattened and became more ellipsoidal following LOR and before ROR, similar to the changes seen with the GABAergic agent administration in the previously discussed experiments (Figure 9).

**Figure 10:**
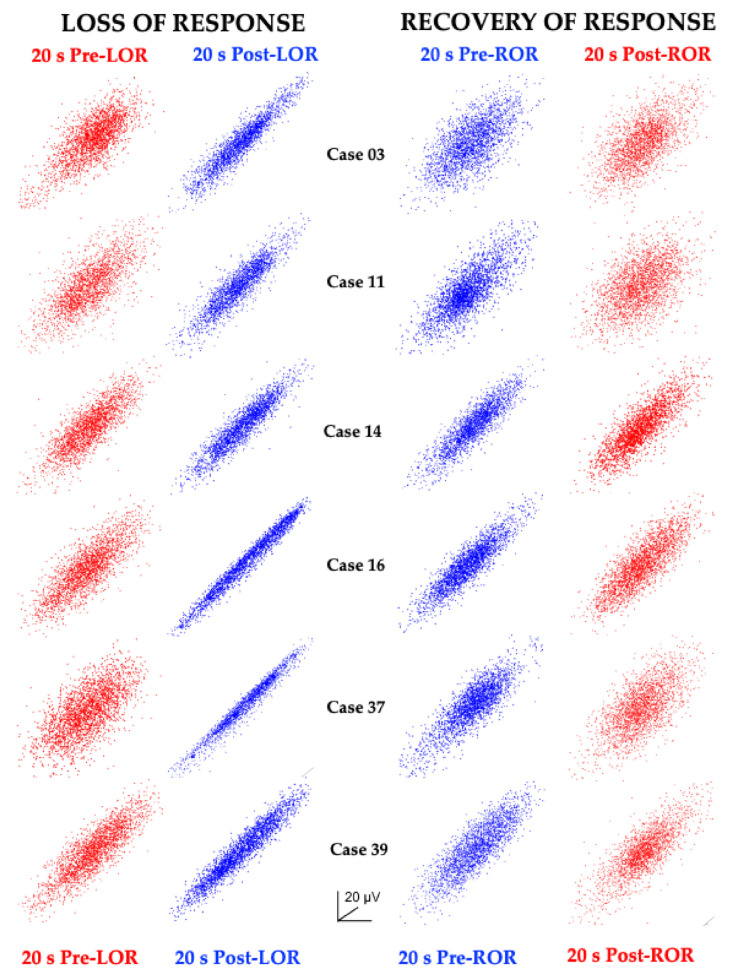
Attractor shapes consistently change across patients anesthetized with remifentanil and nitrous oxide. Attractors generated from 20 s EEG clips before and after loss of response (LOR) and recovery of response (ROR) from six patients show flattening and more ellipsoidal shapes following LOR, and more spherical shapes following ROR consistently across patients anesthetized with remifentanil and nitrous oxide. Awake, responsive states are shown in red, and anesthetized, unresponsive states are shown in blue. Reproduced with permission from Reference [5].

Interestingly, the geometrical attractor shape changes were consistent across subjects before and after loss and recovery of response (Figure 10).

**Figure 11:**
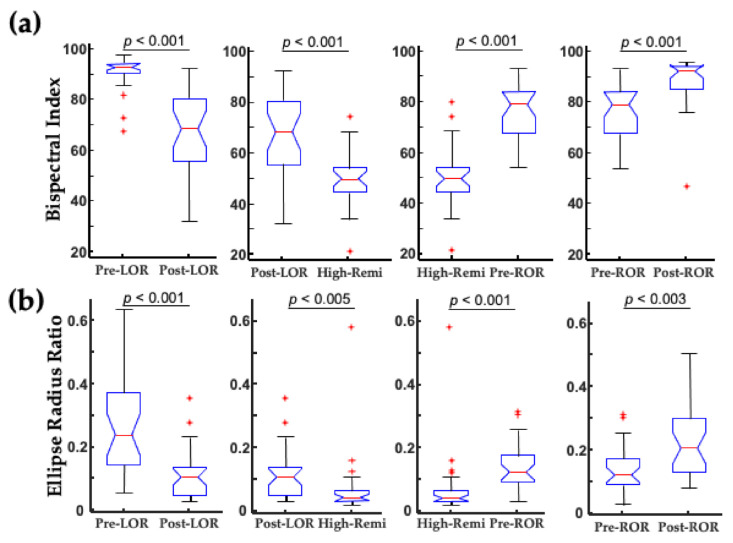
Ellipse radius ratio discriminates clinically relevant states as well as the bispectral index. (**a**) The bispectral index (BIS) significantly differs between the clinical loss and recovery of consciousness when a combination of remifentanil and 66% N_2_O is administered. Significant differences in BIS also occur between post-LOR and pre-ROR and deep levels of anesthesia (High-Remi, during which the highest concentration of remifentanil was administered). (**b**) Similar significant differences are observed using the geometric phase-space analysis (ellipse radius ratio). Uncorrected *p*-values are shown here. Box and whisker plots show the median (red line), 25th and 75th quartiles (bottom and top edges of the blue box, respectively), most extreme data points (error bars), and outliers (red plus signs). Adapted with permission from Reference [5].

For this study, we applied the geometric phase–space analysis (ellipse radius ratio, ERR) measure, which showed significant differences before and after LOR and ROR (Figure 11). We also tested whether differences could be seen between post-LOR and pre-ROR and the deepest level of anesthesia when the highest concentrations of remifentanil were administered (High-Remi, Figure 11). The ERR measure showed non-inferiority to the BIS index to distinguish between these clinically relevant time points.

We have thus far demonstrated the utility of complexity measures to capture EEG changes that occur with the anesthetic administration of diverse agents. In addition to the challenges that arise from using multiple agents to achieve balanced anesthesia, patients at the extremes of age present unique challenges to monitoring given structural and metabolic differences.

## 4. Certain Patient Cohorts Provide Additional Challenges Given Physiological and Metabolic Brain Changes

Pediatric (<18 years of age) and geriatric (aged 65 years or older) patients present challenges for EEG monitoring because of age-dependent differences in brain structure and physiology. Neurodevelopmental factors including glucose metabolism, myelination, and synaptogenesis across cortical regions underlie the unique EEG spectral changes that occur with anesthesia in infants [59]. Spectral EEG differences with anesthetic administration are also seen from infancy through young adulthood [60,61]. Given these differences, it is not surprising that pediatric patients present unique monitoring challenges for existing commercial brain monitors [62].

At the other end of the human lifespan, many regulatory systems that maintain homeostatic balances dramatically slow or become impaired, which impedes drug clearance and increased drug sensitivity [63,64]. Additionally, overall power decreases [65], complexity decreases [66], and noise level increases [67] are evident in geriatric brains due to the loss of brain white matter [68] and metabolic rate changes [69]. It is not surprising that geriatric patients also present unique challenges to monitoring anesthesia [70].

**Figure 12:**
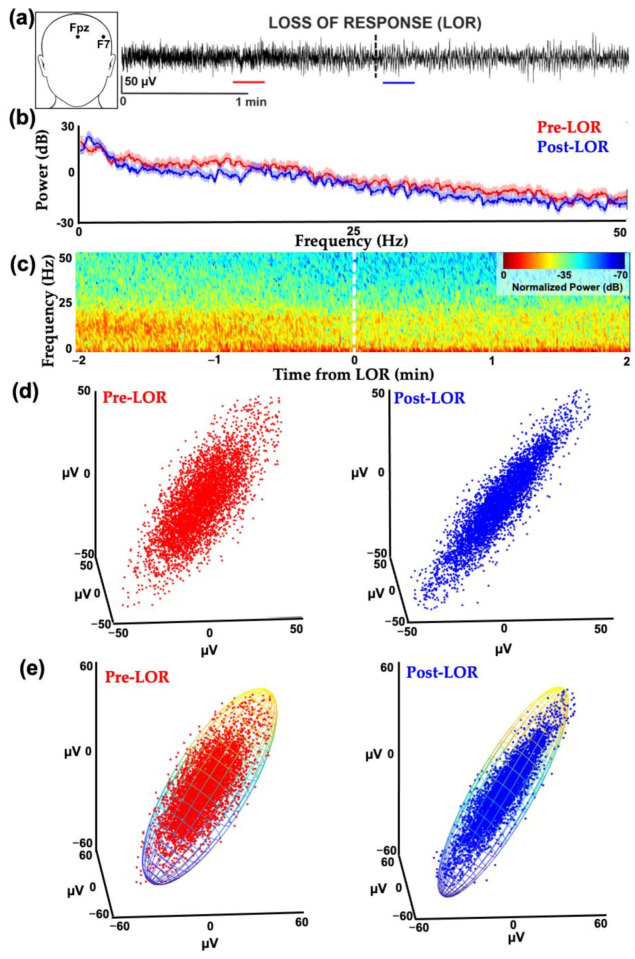
Example of geriatric patient EEG frontal activity from before and after loss of response with fentanyl and propofol. (**a**) EEG activity from electrode location F7 (inset) before (red line) and after (blue line) loss of response (LOR) in geriatric patient anesthetized with a combination of fentanyl and propofol. (**b**) Spectrum from 20 s EEG clip from before (red) and after (blue) LOR. (**c**) Normalized spectrogram of EEG activity starting from 2 min before LOR to 2 min following LOR. For both (**b**) and (**c**), it is difficult to see the spectral differences between before and after LOR, given the overall loss in power in the EEG signal commonly observed in geriatric patients. (**d**) Attractors from 20 s EEG clips from patients before LOR (red) to after LOR (blue). Following LOR, the same geometric shape change occurs where attractors become more ellipsoidal and flatten, as shown in previous examples. (**e**) Same attractors from (**c**), fitted with ellipsoid solid of revolution which was used for subsequent analysis. Reproduced with permission from Reference [6].

We evaluated spectral and complexity measures in EEGs collected from geriatric surgical patients on beta-adrenergic blockades who were anesthetized with a combination of fentanyl and propofol [6,71]. Geriatric patients on beta-adrenergic blockades are more sensitive to the effects of anesthetics and have altered cardiovascular and EEG responses [72,73,74]. We found that spectral and attractor shape changes were evident from before to after LOR in geriatric patients (Figure 12).

**Figure 13:**
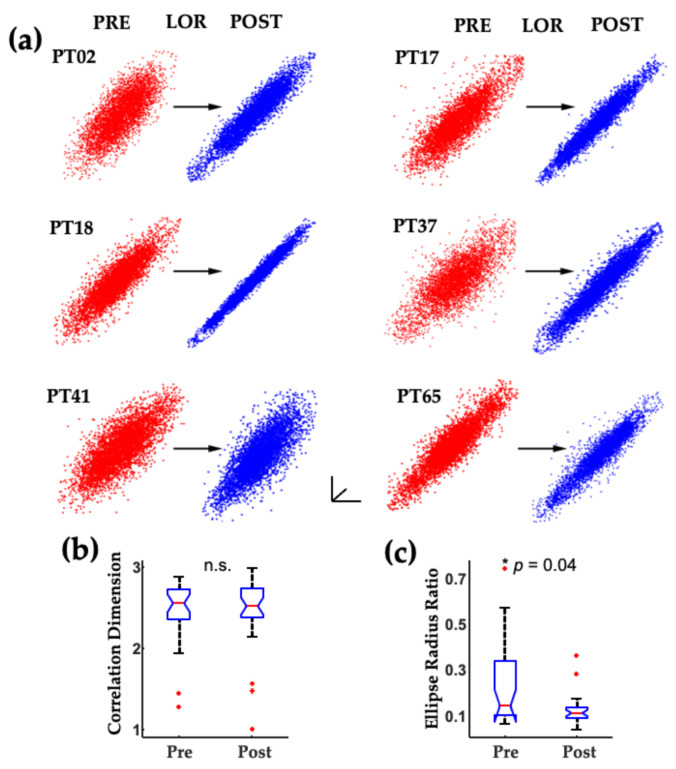
Consistent changes in geriatric patient EEG attractors occur with a loss of response. (**a**) Attractors generated from 20 s clips of EEG activity from before (red) and after (blue) loss of response (LOR) show consistent flattening and more ellipsoidal shapes following LOR. (**b**) Correlation dimension attractor measure did not capture the change in activity following LOR. (**c**) However, the geometric phase–space measure (ellipse radius ratio) significantly decreases following LOR. Attractors are auto-scaled to illustrate the shape changes that occur with LOR. The axes are shown to demonstrate that these are 2D projections of 3D attractors. The * indicates significance for the Holm–Bonferroni corrected *p*-value shown here, n.s. indicates a not significantly different comparison. Adapted with permission from reference [6].

We found that the ERR measure, but not correlation dimension measure, significantly differed between the before and after LOR states (Figure 13).

## 5. Discussion

We have described the unique challenges that arise in anesthesia monitoring, given the diverse molecular targets of anesthetic agents, the practice of combining agents to produce balanced anesthesia, and the challenges presented with brain structure and function in older patients. We have reviewed our previous work, demonstrating that complexity measures that capture the disruption of cortical processing generally show high utility in distinguishing clinically relevant states produced by anesthetics with different molecular mechanisms [5,7], in hard-to-monitor age cohorts [6] in humans and in rodents [4].

A great deal of work has uncovered the disruptions of functional connectivity in the brain from diverse molecular cascades that steal away consciousness [42,43,44,45,52,75]. The loss of functional connectivity imposes regularity, and loss of complexity in EEG signals, which the measures we have tested were able to capture [76,77]. EEG complexity provides a measure of degraded synaptic connections that accumulate with increasing anesthetic concentrations, and increased degradation leads to reduced complexity. As cortico-cortical excitatory synapses begin to fail [76], and circuit timing is slowed by GABA inhibitory postsynaptic current prolongation, complex rhythmic oscillations degrade into slow wave activity [78]. At the loss of consciousness, increased slow wave activity exhibited a corresponding loss of complexity in the EEG signal. Finding commonalities in network level disruption from interacting anesthetic agents that target diverse molecular receptors may lead to a deeper understanding of anesthetic mechanisms and help to generate clinically useful computational tools to serve as biomarkers to monitor anesthetic depth.

## Figures and Tables

**Table 1:** Summary of uses, mechanisms of action, electroencephalogram (EEG) effects, advantages, and disadvantages of all anesthetic agents mentioned in this review.

Anesthetic Agent	Uses	Mechanism of Action	EEG Effects	Advantages	Disadvantages
Propofol	Common general anesthetic. Can be administered at low doses as a sedative.	GABA agonist [8].	Alpha and beta oscillations during sedation. Sleep-like EEG pattern during general anesthesia. Burst suppression to isoelectricity at higher doses [8].	Rapid onset and offset.	Hard-to-monitor without EEG. Can decrease blood pressure and cause breathing difficulties.
Halogenated Ethers (e.g., Sevoflurane)	Common general anesthetic.	Predominately suppresses glutamate-mediated excitation.	Sleep-like EEG pattern during general anesthesia. Burst suppression to isoelectricity at higher doses.	Rapid onset and offset. Easy-to-monitor with MAC values.	May lead to extended burst suppression and peri-operative neurocognitive decline in geriatric patients.
Nitrous Oxide	Supplemental anesthetic agent. Used for analgesia.	NMDA antagonist.	Maintenance of wake-like EEG activity in beta and gamma range [8,9,10].	Rapid onset and offset. Few drug interactions.	Weak anesthetic agent. Increased risk of nausea [11].
Ketamine	Can be used for sedation, as a supplemental anesthetic agent, and for antinociception.	Many molecular targets, including NMDA receptors [1,12].	Beta and gamma oscillations at sedation [8]. Maintenance of wake-like EEG activity.	Bronchodilator, protects patients with reactive airways, preserves spontaneous respirations and airway [12].	Frequent use could cause memory impairment. Increases muscle tone. Airway compromise may occur [12]
Dexmedetomidine	Used as a supplemental anesthetic agent and for analgesia.	Alpha-2-adrenoceptor agonist. Blocks norepinephrine release which activates GABA and other inhibitory projections [8].	Maintenance of wake-like EEG activity.	Anesthetic sparing. Preserves respiratory function and reduces delirium. Cardiovascular sparing [13].	May cause bradycardia, hypotension, hypertension, nausea, and dry mouth [13].
Remifentanil	Used as a supplemental anesthetic agent [14] and for analgesia.	Mu-type-opioid receptor agonist.	Sleep-like EEG pattern during general anesthesia [15].	Rapid onset and offset compared to other opioids. Can decrease overall general anesthesia needs [15].	May increase nausea, respiratory depression, and hypoxia [16].

All agents listed are used clinically in pediatric, adult, and geriatric patients; however, their use is guided by the clinical need and patient comorbidities and medications. This table includes several details about these agents, but is not inclusive. GABA stands for gamma-aminobutyric acid. NMDA stands for N-Methyl-D-aspartate.

## Abbreviations

EEG	Electroencephalogram, electroencephalography
GABA	Gamma-aminobutyric acid
LOR	Loss of response
ROR	Recovery of response
CD	Correlation dimension
ERR	Ellipse radius ratio
N_2_O	Nitrous oxide
High-Remi	Highest blood concentration of remifentanil
BIS	Bispectral index
